# Applications and Developments on the Use of Vibrational Spectroscopy Imaging for the Analysis, Monitoring and Characterisation of Crops and Plants

**DOI:** 10.3390/molecules21060755

**Published:** 2016-06-10

**Authors:** Daniel Cozzolino, Jessica Roberts

**Affiliations:** School of Medical and Applied Sciences, Central Queensland Innovation and Research Precinct (CQIRP), Central Queensland University (CQU), Bruce Highway, North Rockhampton, Qld 4701, Queensland, Australia; j.j.roberts@cqu.edu.au

**Keywords:** crops, plants, infrared, hyperspectral, spectroscopy

## Abstract

The adaptation and use of advanced technologies is an effective and encouraging way to efficiently and reliably characterise crops and plants. Additionally advances in these technologies will improve the information available for agronomists, breeders and plant physiologists in order to develop best management practices in the process and commercialization of agricultural products and commodities. Methods based on vibrational spectroscopy such as near infrared (NIR) spectroscopy using either single spot or hyperspectral measurements are now more available and ready to use than ever before. The main characteristics of these methodologies (high-throughput, non-destructive) have determined a growth in basic and applied research using NIR spectroscopy in many disciplines related with crop and plant sciences. A wide range of studies have demonstrated the ability of NIR spectroscopy to analyse different parameters in crops. Recently the use of hyperspectral imaging techniques have expanded the range of applications in crop and plant sciences. This article provides an overview of applications and developments of NIR hyperspectral image for the analysis, monitoring and characterisation of crops and plants.

## 1. Introduction

Although near infrared (NIR) spectroscopy has often been applied or used for the analysis of different properties in crops and plants, most of these applications rely on spot measurements of the sample [1,2,3,4,5,6]. Nowadays, the availability of hyperspectral cameras and spectrographs have provided exciting new possibilities for the detection of several properties in a wide range of agricultural products and foods [1,2,3,4,5,6]. However, in order to expand and add other analytical possibilities to the analysis and monitoring of several properties in crops and plants, other sensors developed for different or complementary regions of the electromagnetic spectrum (e.g., detection of defects in fruits, composition) need to be used [1,2,3,4,5,6]. The visible (VIS) range have been extensively used to analyse crops, fruits and plants. Conversely, broadband images (e.g., grey-scale and colour images) are generally considered unsuitable to measure quality attributes other than colour because most of the chemical parameters or properties such as carbohydrates, proteins or lipids, are not sensitive to be measured in the VIS range [1,2,3,4,5,6]. On the other hand, modern, spectral imaging technologies, which acquire single or multiple images at selected wavelengths, have been favoured to be used in order to detect specific quality attributes in a wide range of crops and horticultural products [7,8,9].

The application of spectral imaging can be categorized into two main types namely multispectral and hyperspectral imaging [9,10,11,12]. Multispectral imaging techniques acquire spectral images at a few discrete narrow wavebands (the bandwidth may range between 5 and 50 nm) where hyperspectral imaging, acquires several spectral images at many different wavelengths or wavebands over a specific spectral region [9,10,11,12]. While acquisition speed is still an issue in modern instrumentation, focal plane array cameras have been applied to solve this issue [9]. Hyperspectral combines and integrates imaging and spectroscopy in order to acquire both spectral and spatial information from the sample at the same time. This technology has become increasingly suitable and more powerful to examine fresh crops and foods, whose properties and characteristics often vary spatially [10,11,12]. As reviewed by other authors, hyperspectral imaging is commonly implemented in one of the two sensing modes such as push broom or line scanning mode and filter-based imaging mode [10,11,12,13]. Applications of these technologies using in-line scanning mode, the imaging system line scans the moving product items, from which three-dimensional (3D) hyperspectral images, also called hypercube, are collected. In systems using the in filter-based imaging approach, spectral images can be collected from the stationary product items of a sequence of wavebands using either liquid crystal tunable filter (LCTF) or an acousto-optic tunable filter (AOTF) [14,15]. Moreover, filter-based hyperspectral imaging systems need for calibrations that are considered more complex and in general terms are not suitable for online applications [14,15].

In general terms, an ideal hyperspectral imaging system will comprise of a high performance digital camera covering the spectral region of interest, a large dynamic range, a low noise level, and good quantum efficiency [9,11,12]. An essential component of the system is the imaging spectrograph, which disperses line images into different wavelengths [9,11,12]. This component should have an appropriate optical resolution and spectral response efficiency with minimal aberrations [9,11,12]. The availability of fast and relatively cheap diode array spectrometers allows acquiring an NIR spectrum in as little as 50 ms [9,11,12]. These types of instruments have boosted research and development towards a wide range of commercial applications [9,11,12]. However, the widespread use of these technologies in the field (on farm applications) will depend on several factors such as cost and availability of instruments, and the type of application (e.g., on line, continuous collection). In addition, model robustness (e.g., calibration development) in terms of accuracy and precision in relation to the targeted parameter to be analysed, is an important factor to be considered for a specific application [9,11,12]. However, the use of these technologies across the entire food supply chain need to be carefully considered and the limits (constraints) of the technology must be appreciated and any misuse monitored by a spectroscopy specialist. 

This article provides an overview of the applications and developments of vibrational spectroscopy, with special emphasis on the applications of NIR hyperspectral image for the analysis, monitoring and characterisation of crops and plants.

## 2. The Main Drivers on the Use of Vibrational Spectroscopy for Crop and Plant Analysis

In recent years, methods based in hyperspectral image have been incorporated in research projects, in particular several applications have been explored in areas of breeding and agronomy [16]. The main driver behind the incorporation of these technologies are related with the limitation of the current state of NIR spectroscopy to measure the phenotype characteristics in crops and plants [16]. As reported by other researchers, high throughput genotyping has provided plant scientist with fast and inexpensive genomic information [16]. Recent developments in agricultural technology (e.g., robotics, drones) led to an increasing demand for a new era of non-destructive methods of plant analysis in the field rather than in the laboratory [16,17].

Currently several Universities and research organizations around the world are carrying out research and development (R&D) towards technologies that will create a practical tool for a large-scale and real-time monitoring of plants (e.g., fruits, vineyards, orchards, grains) or plant parts (e.g., leaves, grains, fruits) under field conditions [16,17]. This area of R&D is considered one of rapid expansion demanding changes in hardware (e.g., portable and easy to use instruments) and software (e.g., algorithms, mathematical models) in the next 20 years. The use of rapid and non-invasive assessment techniques to fingerprint compositional, physiological and biochemical traits that can be used to improve plant varieties, monitor fertilization, and determine composition has become a new area of R&D [16,17].

## 3. What Is Measured by These Technologies?

It is desirable that methods for plant analysis based on hyperspectral imaging should be rapid, non-destructive, and easy to use. Additionally these technologies needs to be specific, and sensitive to changes in the chemical and physical properties of the plant tissue or sample under analysis. The combination of both spectroscopic and imaging techniques based on NIR, mid infrared (MIR), Raman, fluorescence or VIS spectroscopy are unique as they provide a comprehensive analysis of the sample. These techniques allow further development of monitoring methods that have been used to detect plant diseases, plant stress due to various factors (e.g., temperature, water, nutrients), as well as the determination of other chemical and physical properties in several plants tissues and samples (e.g., fruits, grains, leaves, whole plants). These techniques alone or combined can be used as tools to develop functional databases that can be used in breeding, farm management, environmental assessment, conservation. The integration of different plant disciplines (e.g., physiology, biochemistry, chemistry, *etc.*), algorithms, modelling, and spectroscopy will facilitate the expansion of tools for reliable, rapid and low-cost analysis. These techniques will also enable farmers to maximise sales in existing markets, to target new markets with a differentiated product and to trace and authenticate the origin of foods. In order to overcome several bottleneck issues and to receive the full benefit of the available genomic information, plant phenomics, must integrate several technologies or methods based in photonics, biology, computers, and robotics that will allow the functional characterization of different plant species. In this way, reliable, automatic, multifunctional, and high-throughput phenotyping platforms can be developed to allow crop and plant physiology with modern tools that can be used by plant scientists to gain with new insight into all the aspects of living plants. Several characteristics or properties that have been analysed by these methods or techniques to date include root morphology, leaf morphology, biomass, properties related to yield and yield components, photosynthetic efficiency, and abiotic stress response. 

## 4. Hyperspectral Imaging Applied to Crops and Plants

This section provides with examples on the use of hyperspectral imaging to determine several properties in crops and plants reported in the literature in the last 10 years. Applications include classification and discrimination of cereals, crop species and diseases.

## 5. Applications

In recent years NIR hyperspectral spectroscopy has been evaluated for its ability to identify wheat samples produced in Canada using wavelet texture features [18]. In this study the authors reported that the wavelet texture analysis of the NIR hyperspectral images of bulk wheat kernels is an effective tool for discrimination between wheat classes [18]. The results reported by these authors indicated that the per cent of correct classification ranged between 63% and 100% depending on the classification method (e.g., linear, quadratic or ANN) used [18]. The use of NIR hyperspectral image analysis has also been evaluated to classify single wheat grain samples representing different Australian varieties described as either sound or discoloured by one of the commercially important properties such as black point, field fungi or pink stains [19]. These authors used a separate training (188 grains) and test set (665 grains) and used penalised discriminant analysis as the method of classification in which a simple rule for grain classification was developed [19]. Overall correct classification accuracies of 95% were reported by the authors using spectra collected using the VIS-NIR wavelength range between 420 and 2500 nm (calibration = 97%, validation = 95%). The same authors explored the use of different wavelength ranges in either the VIS and NIR domain such as 420–1000 nm (calibration = 98%, validation = 95%) and 420–700 nm (calibration = 95%, validation = 95%) [19]. These results also indicated that the VIS range of the electromagnetic spectrum might be useful for the classification of wheat samples [19]. Vitreousness is an important grading factor for durum wheat kernels that is associated with protein content [20]. Hyperspectral reflectance images of wheat kernels having different vitreousness characteristics were collected using the wavelength range between 650–1100 nm [20]. A discrimination method based on latent variables extracted using partial least squares (PLS) allowed satisfactory discrimination results (100% correct separation between vitreous and non-vitreous kernels) and a correct classification rate of up to 94% for the discrimination of total and partial starchy kernel classes [20].

Most authors have suggested that the NIR spectral differences between samples for specific traits was a result of interactions between different chemical (e.g., amylose content, lipid-amylose interactions, protein) and physical (e.g., scattering) properties of the endosperm [21,22]. In general, the vast majority of classification models reported have applied principal component analysis (PCA) or artificial neural networks (ANN), where ANN models proved to perform better than PCA with a Mahalanobis distance classifier [21,22]. Differentiation of wheat classes is one of the important challenges to the Canadian grain industry. Although some wheat varieties could appear very similar under visual inspection, both grain composition (e.g., protein, starch) as well as the end product quality can differ significantly [21,22]. 

NIR hyperspectral imaging was used to develop classification models to differentiate wheat classes grown in western Canada [21,22]. Wheat bulk samples were scanned in the wavelength region between 960 and 1700 nm at 10 nm intervals using an indium gallium arsenide (InGaAs) NIR camera [21,22]. Classification accuracies reported for each of the groups analysed were 100% in classifying Canada prairie spring red (CPSR), western red winter (CWRW), and western soft white spring (CWSWS) wheat classes and higher than 94% for the other wheat classes analysed including western extra strong (CWES), western hard white spring (CWHWS), western red spring (CWRS), prairie spring white (CPSW) and western amber durum (CWAD) using linear discriminant analysis (LDA) [21,22]. These authors also explored the use of quadratic discriminant analysis (QDA) with a leave-one-out cross-validation method, reporting classification accuracies of higher than 86% for all wheat classes. The reported results by these authors indicated that for models developed using ANN the classification accuracies were above 90% for the independent validation set using the three-layer standard and Wardnet back-propagation neural network architectures [21,22]. The authors also concluded that one of the major limitations of this technique is the production of a large volume of information that requires appropriate data processing techniques to interpret the results accurately [21,22]. The same authors also highlighted the fact that proper calibration methods are needed to remove the inherent and external noise in the system during imaging [21,22,23].

A NIR hyperspectral imaging system was used to identify rice seed samples. Classification models were developed using PLS discriminant analysis (DA), soft independent modelling of class analogy (SIMCA), K-Nearest Neighbors (KNN), support vector machine (SVM), and random forest (RF) techniques [24]. Spectra between 1039 nm and 1612 nm were used in their entirety to build classification models. PLS-DA and KNN models produced over 80% classification accuracy while SIMCA, SVM and RF models generated 100% classification accuracy in both the calibration and prediction sample sets [24]. Overall results indicated that hyperspectral imaging could be used for rice seed cultivar identification and that RF is an effective classification technique [24]. The use of NIR hyperspectral imaging and hyperspectral image analysis for distinguishing among different maize kernels (e.g., hard, intermediate or soft) from inbred lines was reported by the authors [25]. Images were obtained from two sets kernels using a spectral dimensions MatrixNIR camera (960–1662 nm) and the SisuChema SWIR (short wave infrared) hyperspectral pushbroom imaging system (1000–2498 nm) [25]. In this study, PCA was used as tool to remove background, bad pixels and shading from the hyperspectral images. These authors reported that using the cleaned images, PCA could be used effectively to find histological classes including glassy (hard) and floury (soft) endosperm [25]. PCA illustrated a distinct difference between glassy and floury endosperm along principal component (PC) three on the MatrixNIR and PC two on the SisuChema with two distinguishable clusters. Discrimination results achieved using PLS-DA, based on the images collected the MatrixNIR (12 kernels), resulted in root mean square error of prediction (RMSEP) value of 0.18. The same RMSEP (0.18) was reported using images from 24 kernels. On the other hand, the authors reported a RMSEP of 0.29 using the SisuChema system [25]. The reproducible results obtained with the different data sets indicate that the method proposed in this paper has a real potential for future classification uses [25].

In order to identify the purity of waxy corn a system based in hyperspectral imaging was developed using the combined spectral, morphological, and textural features extracted from VIS and NIR hyperspectral images [26]. These authors applied some pre-processing techniques, in order to reduce the dimensions of the spectral dataset, where they constructed spectral feature vectors using the successive projections algorithm (SPA) [26]. Five morphological features (area, circularity, aspect ratio, roundness, and solidity) and eight texture features (energy, contrast, correlation, entropy and their standard deviations) were extracted as descriptive appearance characters from every corn kernel [26]. Both SVM and PLS-DA were used to build classification models for seed variety classification based on different groups of features [26]. Results reported by these authors demonstrated that, by combining spectral and appearance characteristics, better classification results were obtained [26]. Recognition accuracy achieved by the SVM model (98.2% and 96.3% for germ side and endosperm side, respectively) were better than those reported using PLS-DA as a classification tool [26].

The classification and identification of grape varieties is achieved by the visual inspection of the vines (ampelometry) or by the use of genetic analysis [27]. These authors developed a simple and automatic method of classification of grapevine varieties using data derived from leaf spectroscopy [27]. The method reported by these authors consists of a classifier based on PLS-DA among grapevine varieties using a hyperspectral image of a leaf measured in reflectance mode [27]. The hyperspectral images were collected using a camera with 1040 wavelength bands operating between 380 nm and 1028 nm [27]. Thus, the classifier was created using 300 leaves, 100 of each of the varieties *Vitis vinifera* L., *Tempranillo*, *Grenache* and *Cabernet Sauvignon* [27]. The authors used Monte-Carlo cross-validation to validate the classifier’s performance for the three varieties (correct classification > 92%) [27].

Hyperspectral images of intact grapes harvested during ripening were recorded using a NIR hyperspectral imaging system (900–1700 nm) [28]. Spectral images were correlated with grape skin total phenolic concentration, sugars and acidity using MPLS regression, and different spectral pre-treatments [28]. The calibration models developed using red and white wine grape samples were coefficient of determination (R^2^) and the standard error in cross validation (SECV) of 0.89 and 1.23 mg·g^−1^ for total phenolic concentration, 0.99 and 1.37 for sugars, 0.98 and 3.88 g·L^−1^ for total acidity and for pH 0.94 and 0.12 [28]. Hyperspectral imaging was reported for the determination of phenolic compounds (total anthocyanins) in the skins of *Cabernet Sauvignon* grapes produced in Shaanxi province (China) [29]. In this study images derived from 60 groups of grape samples were acquired using a NIR hyperspectral camera (900–1700 nm) [29]. The anthocyanin content of the grape skin was determined with a pH-differential method as reference method [29]. The grape berry regions of hyperspectral images were extracted as region of interest (ROI) in which its average spectrum was calculated [29]. Different pre-processing methods were used to improve the signal noise ratio (SNR) including Savitzky-Golay smoothing, normalization and multiple scatter correction (MSC) [29]. A prediction model was established for determining anthocyanin content using PLS regression, least squares, SVM and BPNN [29]. A R^2^ of 0.91 and RMSEP of 0.38 for TSS (°Brix) using a BPNN model was reported by the authors [29]. The potential of NIR hyperspectral imaging to determine anthocyanins in intact grapes has been also reported [30]. The hyperspectral images of intact grapes during ripening were collected using NIR hyperspectral imaging covering the spectral range between 900 and 1700 nm [30]. Calibrations were developed using MPLS as an algorithm, where a coefficient of correlation (R) of 0.86 and SEP values of 2.62 and 3.05 mg·g^−1^ for the measurement of non-acylated and total anthocyanins in wine grape skin samples were reported by the authors [30]. Hyperspectral imaging has been explored as an alternative technology to characterize grape seeds according to their chemical composition (not specified by the authors) and variety [31]. Non-destructive characterization of grape seeds with regards to their variety and stage of maturation was reported by the authors [31]. Seed samples from two red grape varieties (*Tempranillo* and *Syrah*) and one white variety (*Zalema*), sourced from two soil types were analysed. The NIR reflectance spectra were analysed using PLS and PCA methods [31]. The authors reported a reliable methodology for predicting the stage of maturation and it was also possible to distinguish the variety of grape and the type of vineyard soil from hyperspectral images of grape seeds, classification rates between 96% to 100% were reported [31].

## 6. The Importance of Hyperspectral in the Detection and Monitoring of Diseases in Crops

Hyperspectral image techniques have been also evaluated for the detection and monitoring of different issues related with crop health, diseases, insect contamination and stress as these applications are of importance for further developments in precision agriculture [32,33,34]. Hyperspectral imaging was used to monitor the disease severity of wheat leaves under stress caused by the infestation by powdery mildew [35]. These authors conducted artificial inoculation experiment of wheat powdery mildew to test winter wheat leaf’s spectral reflectance under powdery mildew with different severity degree in different growth phases using hyper-spectrometer [35]. Using artificially inoculation method, the authors measured the spectra of wheat leaves of different varieties at different levels of incidence and growth stages, and investigated the disease severity of each leaf [35]. The relationships between the NIR data and the disease severity caused by powdery mildew was analysed using factor analysis-back propagation neural network (FA-BPNN) method, and evaluated for its fitting accuracy and potential applicability [35]. The results reported by these authors indicated that the spectra of the leaves obtained in the VIS range (350–750) increased with the aggravation of disease severity as consequence of the infestation by powdery mildew, while spectral reflectance decreased in the NIR bands (760–1050 nm) [35]. The authors also showed that the best two-band vegetation index that was correlated with wheat powdery mildew between 400 and 1000 nm wavelength was located in band combination of 605–630 nm and 520–550 nm, 645–690 nm and 710–1000 nm for the ratio index, and in band combination of 650–685 and 710–1000 nm for the normalization index [35]. These results indicated that the BPNN simulation could greatly improve the estimation accuracy of disease severity of wheat leaf powdery mildew [35]. Reflectance measurements were used to detect wheat yellow rust disease severity where calibration models were developed using PLS, BP neural network using seven hyperspectral vegetation indices which having significant relationship with the occurrence of disease and vegetation index (PRI) were adopted to build a feasible regression model for detecting the disease severity [36]. Different wheat varieties were planted in field and stripe rust was caused by artificial inoculation [36]. The results reported by these authors showed that different combinations of wheat varieties had the similar reflectance variation at different disease index [36]. Models for the estimation of the disease index were developed based on the canopy reflectance at 690 and 850 nm [36]. These results indicated that hyperspectral remote sensing method developed by these authors can predict the disease index associated with rust in wheat where the results were not influenced by the different combinations of wheat varieties [36]. A real time remote sensing system was developed as a rapid and field based method to identify healthy and infected plants at an early stage of disease development [37]. In this study, the authors inoculated tobacco plants with the black-shank disease and images collected in the VI-NIR range [37]. The images acquired were analysed using PCA in order to find the optimal wavelengths for determining and evaluating the level of damage by the black-shank fungus [37]. The results reported by these authors indicated that the spectral reflectance decreases significantly with the increasing severity level in both the VIS and NIR wavelength ranges [37].

In another experiment, wheat plants were artificially infested with wheat stem sawflies, and hyperspectral images (reflectance range from 402.8–838.7 nm) were collected from leaves of infested and non-infested plants [38]. The use of quantitative tools in spatial ecology such as variogram analysis where used by these authors to suggest the need for further research into its use in remote sensing where biotic stress is the main target [38]. Reflectance spectra of cotton leaves infected with *Verticillium* were measured in cotton disease nursery and field in different growth phases [39]. The results reported by these authors indicated that the correlations were best significant between pigments such as chlorophyll contents of leaves and spectral reflectance in VIS range [39]. 

The Russian wheat aphid (RWA, *Diuraphis noxia*) is an insect pest that causes damage to wheat (*Triticum aestivum* L.) [40]. Percent surface reflectance from uninfected wheat was lower in the VIS and higher in the NIR wavelength range of the spectrum when compared with RWA-infested wheat [40]. The overall classification accuracies were higher than 89% for damage detection were achieved. These results indicated that hyperspectral can be effectively used for accurate detection and quantification of RWA infestation in wheat for site-specific aphid management [40].

## 7. Summary

The adaptation and use of advanced technologies such as hyperspectral imaging is a promising way forward to efficiently and reliably improve agronomically important characteristics in the breeding process of several crops and plant species. This same technologies will allow to have better tools to improve management farming practices, as well as move forward to best management practices in the process and commercialization of agricultural products and commodities.

Infrared (NIR and MIR) technologies have been introduced as fingerprinting techniques within agriculture and food sector. Several studies have been showed their role in the analysis of plant to plant interactions, where global metabolite changes associated with abiotic and biotic perturbations and interactions can be measured simultaneously. Spectra collected in the NIR and MIR regions of the electromagnetic spectrum provide with the so called fingerprint of a given sample, which with the aid of multivariate data analysis techniques (e.g., principal component analysis or discriminant analysis), can be used to elucidate particular compositional characteristics not easily detected by traditional targeted chemical analysis. The benefits of this type of instrumental methods over the traditional methods currently in use are the analytical speed and of easy operating. Moreover, IR is a non-destructive technique which requires minimal or zero sample preparation. The potential savings, reduction of analysis time and cost, and the environmentally friendly nature of the technology has positioned IR spectroscopy as a very attractive technique with a bright future in the arena for on farm measurements of plant properties. However, the use of these technologies require the use of multivariate data analysis methods and techniques in order to analyse and interpret the data generated.

The development of hyper spectral imaging, micro spectroscopy and new algorithms (topics not covered in this report) will place IR spectroscopy as one of the most useful tools in plant studies and as the preferred tool in field technology in the near future.
